# PCDA/ZnO Organic–Inorganic Hybrid Photoanode for Efficient Photoelectrochemical Solar Water Splitting

**DOI:** 10.3390/ma17174259

**Published:** 2024-08-28

**Authors:** Nursalim Akhmetzhanov, Mao Zhang, Dongyun Lee, Yoon-Hwae Hwang

**Affiliations:** 1 Department of Nano Fusion Technology & BK FOUR Nanoconvergence Technology Division, Pusan National University, Busan 46241, Republic of Korea; 2 Department of Nanoenergy Engineering, Pusan National University, Busan 46241, Republic of Korea

**Keywords:** photoelectrochemical water splitting, photoanode, ZnO, poly-10,12-pentacosadiynic acid, hydrogen energy

## Abstract

In this study, we developed well-aligned ZnO nanoflowers coated with poly-10,12-pentacosadiyonic acid (p-PCDA@ZnO) and modified with Pt nanoparticle (Pt/p-PCDA@ZnO) hybrid photoanodes for highly efficient photoelectrochemical (PEC) water splitting. The scanning electron microscope (SEM) image shows that thin films of the p-PCDA layer were well coated on the ZnO nanoflowers and that Pt nanoparticles were on it. The photoelectrochemical characterizations were made under simulated solar irradiation AM 1.5. The current density of the p-PCDA@ZnO and the Pt/p- PCDA@ZnO was 0.227 mA/cm^2^ and 0.305 mA/cm^2^, respectively, and these values were three times and four times higher compared to the 0.071 mA/cm^2^ of the bare ZnO nanoflowers. The UV–visible spectrum showed that the absorbance of coated p-PCDA films was extended in visible light region, which agrees with the enhanced PEC data for p-PCDA@ZnO. Also, adding Pt nanoparticles on top of the films as co-catalysts enhanced the PEC performance of Pt/p-PCDA@ZnO further. This indicates that Pt/p- PCDA@ZnO has a great potential to be implemented in solar water splitting.

## 1. Introduction

In the last couple of decades, green and sustainable sources of energy were only used for global causes such as climate change, but nowadays, alternative approaches are becoming more and more economically desirable. Globally, most economies are still dependent on highly concentrated oil and gas basins in certain regions. So, research into renewable sources of energy is more intense than it was before. Renewables have the key advantage of being decentralized. Even single households can maintain their own solar panels or wind turbines. Solar water splitting (SWS) is one type of a sustainable, zero-emission energy source that has the potential to be widely employed. The two main chemical reactions in SWS are the oxygen evolution reaction (OER) and the hydrogen evolution reaction (HER). The SWS system was first realized by K. Honda and A. Fujishima in 1972 [1]. They created a photoelectrochemical cell based on TiO_2_ that was able to evaluate oxygen from water under solar irradiation. Fujushima et al. proved H_2_ evolution under solar irradiation on the Pt/TiO_2_ electrode [2]. At that time, only a limited number of electrodes for SWS were available, including TiO_2_, ZnO, CdS, InP, and MoS_2_ [1,3], meaning that hydrogen production by SWS was far from being a competitor to the electricity produced by conventional energy sources. Recently, the use of hydrogen as green energy by the industrial sector has been continuously increasing, and the abundant source of water means that SWS is still a hot topic [4,5]. For example, several automobile companies are already producing hydrogen-based vehicles, boosting interest in hydrogen production [6]. 

SWS is an economically and eco-friendly attractive option for sustainable growth; however, SWS has some drawbacks that need to be solved before its implementation on a large scale. These are a low solar-to-hydrogen (STH) conversion efficiency, a low-cost effectiveness [7], robustness [8], and its bandgap tenability in electrode materials [9]. To increase STH conversion efficiency, various types of electrodes and catalysts are continuously being proposed by many researchers [8,10]. Catalysts are an effective tool to lower the activation energy in chemical reactions so as to improve STH conversion efficiency [8]. Also, developing new materials [4] and introducing nano-structured materials for electrodes are essential for the development of SWS technology [10,11]. Unfortunately, even though it has been several decades since Honda and Fujishima developed SWS, not many suitable semiconductors for PEC applications have become available [12,13,14]. Most of those semiconductors are either too rare or not durable for electrolytes in SWS. The commonly used materials for electrodes are TiO_2_ and ZnO due to their having bandgaps near 3.2 eV, which is suitable for water decomposition in an ambient condition [1]. In addition, both TiO_2_ and ZnO are stable for a prolonged operation and are abundant enough to be implemented on an industrial scale [15]. However, the bandgap of those two materials corresponds to the UV range; it can capture only ~4% of solar irradiation. To improve this, photosensitizers are used to capture the visible and NIR ranges of solar irradiation and transfer excited electrons into the conduction band of the main electrode [16,17]. Hou et al. also found that by coating ZnO with branched polyethylenimine (BPEI), the current density reached 0.25 mA/cm^2^ compared to 0.18 mA/cm^2^ of bare ZnO [18]. In another work, current density improved by up to 67% by adding PEDOT:PSS film onto CdS_2_/ZnO/WO_2_ photoanode [19]. 

In this study, we tried to extend the solar irradiation range up to the NIR range by introducing poly-10,12-pentacosadiyonic acid (p-PCDA) as a self-assembled monolayer (SAM), which can act as a photosensitizer. In addition, ZnO nanoflowers were also used to develop a new design of hybrid SAM organic–inorganic photoelectrode for PEC applications. Moreover, a Pt co-catalyst was added to make Pt/p-PCDA@ZnO assembly to improve the overall performance of SWS process. We found that the current densities of p-PCDA@ZnO and PCDA@ZnO were 0.227 mA/cm^2^ and 0.305 mA/cm^2^, respectively, and that these values are three and four times higher compared to 0.071 mA/cm^2^ of bare ZnO nanoflowers. Also, it was observed in the UV–visible spectrum that the absorbance of coated p-PCDA films was extended into the visible range. Therefore, we can confirm the effectiveness of the Pt/p- PCDA@ZnO electrode for a high performance of PEC in SWS. 

## 2. Experimental Procedure

### 2.1. ZnO Nanoflower Synthesis

The FTO glass was used for a substrate and was washed with acetone (99.5%), ethyl alcohol (95%), and DI water in a sonication bath for 10 min each before use. The ZnO film was deposited onto washed 1 × 1 cm^2^ of FTO glass via RF sputtering with the ZnO (99.99%) target. The deposition chamber was depressurized down to 1.0 × 10^−6^ Torr. During ZnO deposition, concentrations of Ar and O_2_ gases were controlled with a pressure of 5 mTorr. The power output and the frequency of RF were 50 W and 13.56 MHz, respectively. Deposition was conducted at 6 nm/min. rate with 200 nm of total ZnO layer thickness. Afterwards, it was used as a seed layer for further synthesis of ZnO nanoflowers. The seed layer was coated with a 250 nm thick PMMA e-beam resistive layer. The PMMA layer was treated with e-beam lithography to make 300 nm diameter holes with a 5 µm separation distance. The hydrothermal method was used for the growth of ZnO nanoflowers on the seed layer. A solution of HMTA (ACS reagent, ≥99.0%, Sigma-Aldrich, St. Louis, MO, USA) and Zn(NO_3_)_2_•6H_2_O (reagent grade, 98%, Sigma-Aldrich) at a concentration of 30 mM was used for the hydrothermal growth of the ZnO nanoflowers. The reaction was conducted in a convection oven at 80 °C for 2 h. The more detailed growth process can be found in the References Section [20].

### 2.2. Hybrid Photoanode Fabrication

**Figure 1:**
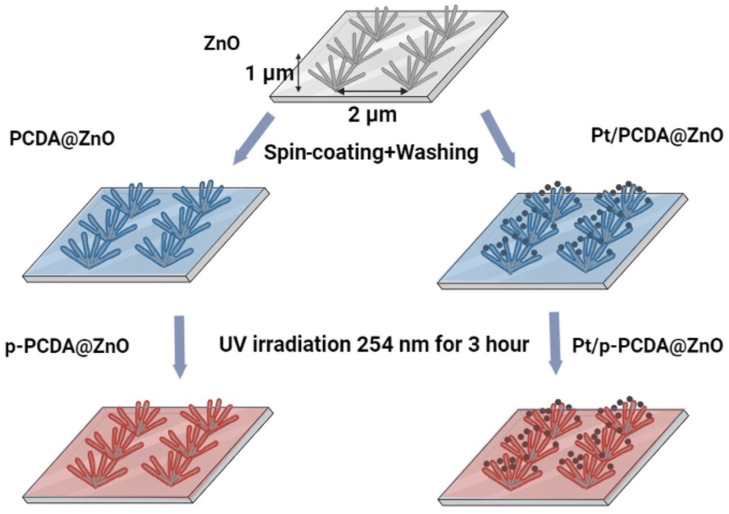
Schematics of patterned ZnO nanoflowers coated with poly-10,12-pentacosadiyonic acid (p-PCDA@ZnO) and Pt/p-PCDA@ZnO photoanodes synthesis route.

The schematic explanation of fabricating p-PCDA@ZnO and Pt/p-PCDA@ZnO hybrid photoanodes is shown in Figure 1. 10,12-pentacosadiynoic acid (p-PCDA, ≥97.0%, Sigma Aldrich) was dissolved in acetone (99.5%) with a concentration of 1 mg/mL to prepare the p-PCDA@ZnO photoanode. This solution was filtered with 0.45 μm of a PTFE-D syringe filter (Hyundai micro, Seoul, Republic of Korea) and then spread onto the ZnO nanoflowers via spin-coating at 1000 rpm for 30 s followed by washing with ethyl alcohol (95%). The spin-coating method was used for an even distribution of PCDA. There was no other treatment needed since PCDA has carboxylic groups that have an affinity with ZnO. This affinity originates from the O-Zn mono, bi-dentate, or bridging interaction mechanism [21]. Afterwards, washing with ethanol was necessary to prevent the deposition of a thick layer of PCDA and to ensure the formation of a monolayer. This process was repeated four times. After monolayer deposition, photopolymerization of PCDA was conducted with a UV lamp (ENF- 240C/FE, Spectro-UV, Farmingdale, NY, USA) for 180 min. at 254 nm [22]. The UV light irradiation forces pairs of triple bonds to break and interact with neighboring molecules to form conjugated polymers [23]. According to Kantha et al., macrocyclic diacetylene photopolymerization was completed in 3 h at 254 nm UV irradiation [24]. Therefore, photopolymerization of the PCDA monolayer was conducted under the same conditions.

To prepare the Pt/p-PCDA@ZnO photoanode, Pt nanoparticles (70 nm, 0.05 mg/mL, citrate functionalized Pt into a 2 mM aqueous sodium citrate buffer, Sigma-Aldrich) were centrifuged and transferred into the PCDA/acetone solution. After mixing, the combined solution was filtered through 0.45 μm of a PTFE-D syringe filter (Hyundai micro). The resultant solution was coated onto the ZnO nanoflowers, and the photopolymerization process of Pt/p-PCDA@ZnO was the same as that of p-PCDA@ZnO.

### 2.3. Characterizations

The morphology of the ZnO nanoflower and p-PCDA@ZnO and Pt/p-PCDA@ZnO photoanodes was investigated with a high-resolution low-voltage scanning electron microscope (HRLV SEM: JEOL JSM-7900F, Tokyo, Japan) to confirm whether the nanostructure and the film formation were successful. The photoelectrochemical (PEC) characterization system, which consists of the reference electrode (Ag/AgCl, saturated in NaCl), counter-electrode (Pt-mesh), and working electrode (ZnO, p-PCDA@ZnO, and Pt/p-PCDA@ZnO), was used to measure the PEC performances of hybrid photoanodes. Linear sweep voltammetry (LSV) and chronoamperometry measurements were conducted via potentiostat (IviumStat, IVIUM Technologies, Eindhoven, The Netherlands) under a solar simulator (ABET Technologies, Milford, CT, USA). The solar simulator provided illumination at AM1.5 and a power of 100 mW/cm^2^. The LSV was conducted to measure current density at “dark” and “light” modes, from 0 V to 2.3 V with a 10 mV voltage step and 50 mV/s scan rate. The chronoamperometry was conducted to see both the stability of a photoanode and its response to different illumination conditions. In the chronoamperometry method, chopping light current density was measured at fixed 1.23 V (water splitting potential) for a total of 100 s with 10 s intervals. The current density was calculated according to the Nernst equation for a Ag/AgCl electrode with a correction to 10.5 pH and with respect to the area that contained ZnO nanoflowers on the FTO glasses. Electrochemical impedance spectroscopy (EIS: Gamry Interface 1000, Warminster, PA, USA) was used to analyze charge transfer mobility at the interfaces under the solar simulator. A UV–visible spectrometer (Thermofisher Scientific, Waltham, MA, USA, EVO300) was used to measure the absorbance of photoanodes.

## 3. Results and Discussion 

### 3.1. HRLV SEM Images and XRD of Photoanodes

**Figure 2:**
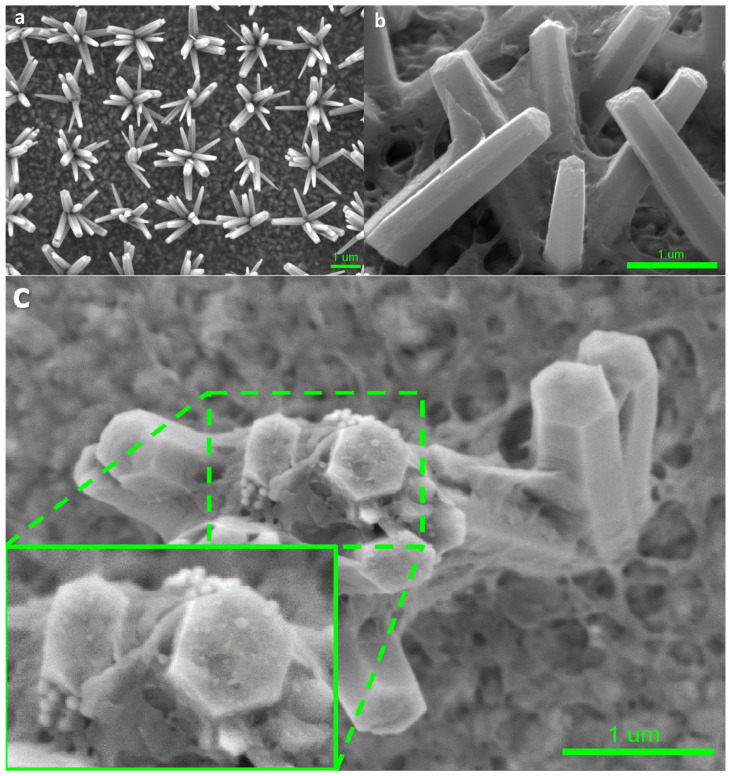
HRLV SEM images of (**a**) bare ZnO nanoflowers on the FTO glass, (**b**) p-PCDA-coated ZnO nanoflowers (p-PCDA@ZnO), and (**c**) Pt nanoparticles on p-PCDA@ZnO film and the enlarged SEM image. In the enlarged image, Pt nanoparticles on top of the ZnO nanorod in the nanoflowers can be clearly seen.

Figure 2a–c show high-resolution low-voltage (HRLV) scanning electron microscopy (SEM) images of the bare ZnO nanoflowers on the FTO glass, and the p-PCDA@ZnO and Pt/p-PCDA@ZnO photoanodes. When we grow vertically aligned ZnO nanorods, usually the population density of ZnO nanorods is high; hence, light illuminates mostly the tips of the ZnO nanorods. On the other hand, the ZnO nanoflowers’ population density allows its tilted rods to be exposed to the light. Therefore, the photosensitizing effect in ZnO nanoflowers can be more enhanced compared to vertically grown ZnO nanorods. The cross-section SEM image of the p-PCDA film-coated ZnO nanorods is shown in Figure S1. As we can clearly observe in this figure, the polymer is coating ZnO and light struggles to penetrate the highly populated ZnO nanorods. Therefore, the ZnO nanorod structure is not suitable for efficient photosensitizing film coating. However, as shown in Figure 2a, bare ZnO nanoflowers are evenly distributed with a distance of 2 μm, and there is no overlapping of individual ZnO nanorods. Hence, the nanoflower structure can have a much larger exposed surface area to light compared with that of a nanorod structure. Figure 2b shows the morphology of p-PCDA film on the ZnO nanoflowers (p-PCDA@ZnO). The thickness of the p-PCDA film throughout the nanoflower structure is on average around 50 nm and can vary from 21 nm to 75 nm (Figure S2a,b).

Figure 2c is the top-view of one Pt/p-PCDA@ZnO nanoflower. We can observe well-coated p-PCDA films on the ZnO nanoflowers with a catalytic amount of Pt nanoparticles on the top facet of the ZnO nanorods in the nanoflower structure. Figure S2c shows that the size of the Pt nanoparticles varies from 55 nm to 68 nm and is comparable to that declared by the manufacturer (~70 nm). This confirms the successful deposition of Pt nanoparticles on the p-PCDA@ZnO hybrid. In the enlarged inset image in Figure 2c, a small amount of agglomerated Pt nanoparticles can be found on the top facet of the p-PCDA@ZnO, which could have happened during Pt nanoparticles preparation. Usually, the citrate buffer is used in commercial Pt nanoparticles (70 nm, 0.05 mg/mL, citrate functionalized Pt into 2 mM aq. sodium citrate buffer, Sigma-Aldrich). Indeed, a formation of curd-like precipitation was observed in some studies, presumably due to citrate condensation with the PCDA in the acetone media [25]. Therefore, a centrifuge process was necessary to extract the Pt nanoparticles from the Pt-functionalized solution. Extracted Pt nanoparticles were mixed with PCDA in an acetone solution and no precipitation of citrate was observed. The resultant solution was evenly spread via spin-coating. Even though during the centrifugation, agglomeration of Pt nanoparticles can happen, we can assume that the Pt nanoparticles are distributed evenly throughout the sample surface in the spin-coating process.

**Figure 3:**
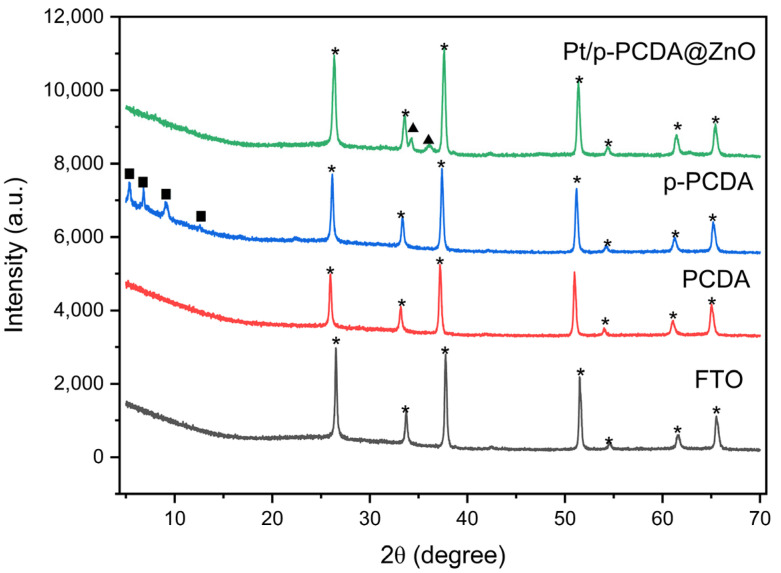
The XRD data of FTO, PCDA, p-PCDA, and Pt/p-PCDA@ZnO. The peaks marked by stars, solid squares and solid triangles originate from FTO glass, p-PCDA polymer film and ZnO nanoflowers.

Figure 3 shows an XRD spectra of FTO, PCDA, p-PCDA, and Pt/p-PCDA@ZnO. The XRD spectrum of FTO has distinctive peaks at 2θ of 26.5°, 33.7°, 37.7°, 51.5°, 54.6°, 61.6°, and 65.5°, which are related to the (110), (101), (200), (211), (220), (310), and (301) planes of FTO, respectively [26]. In the blue spectrum, distinct peaks can be observed at 5.4°, 6.8°, 9.1°, and 12.56°. Those peaks must be due to the p-PCDA polymer film. However, in the Pt/p-PCDA@ZnO spectrum, polymer-related peaks below 15° are not observed. This is because the p-PCDA film in the hybrid is too thin to be detectable. On the other hand, ZnO distinct peaks at 34.3° and 36.0° can be clearly seen. Those are coming from the ZnO nanoflowers (002) and the (101) plane [27].

### 3.2. Photoelectrochemical Characterization

**Figure 4:**
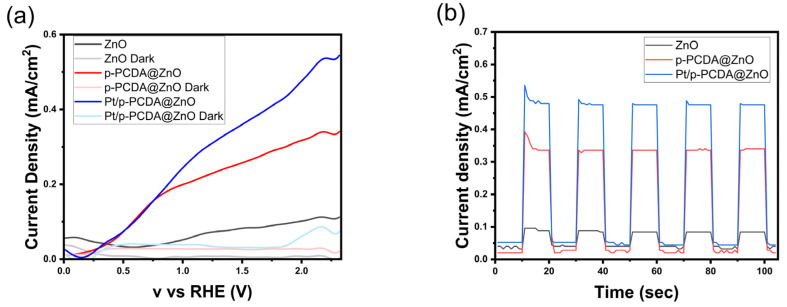
Photoelectrochemical test under solar irradiation of AM 1.5 (100 mW/cm^2^) for three different photoanodes: ZnO, p-PCDA@ZnO, and Pt/p-PCDA@ZnO; (**a**) LSV curve with current densities at “light” and “dark” modes and (**b**) chronoamperometry under alternating “light” and “dark” modes 10 s each.

Figure 4 shows the photoelectrochemical analysis of p-PCDA@ZnO and Pt/p- PCDA@ZnO photoanodes. Figure 4a shows linear sweep voltammetry (LSV) analysis under the solar simulation of AM 1.5 (100 mW/cm^2^) and dark current. Under simulated solar irradiation, the p-PCDA@ZnO and Pt/p-PCDA@ZnO photoanodes show a current density of 0.227 mA/cm^2^ and 0.305 mA/cm^2^, respectively. It is a significant enhancement in the current density compared to the 0.071 mA/cm^2^ of the bare ZnO nanoflowers. As we expected, the output of the Pt/p-PCDA@ZnO photoanode is the highest. The p-PCDA film layer acts as a photosensitizer and transfers the energy absorbed from visible light that a bare ZnO is unable to absorb, and the Pt nanoparticles act as a co-catalyst. Moreover, even at lower voltages, the p-PCDA film still can provide a current since the polymer’s absorption bands are located at longer wavelengths. However, if not optimized, the p-PCDA film thickness can worsen the output of the sample. Figure S4 shows that when the film is thinner (two rounds of coating), the photosensitizing effect is minor. On the other hand, if the film is thicker (six rounds of coating), the travel distance of a charge is also increased, and the charge recombination rate upon charge traveling results in a lower output [28]. Therefore, a sample with four rounds of coating was selected.

The chronoamperometry data are shown in Figure 4b. It shows the current density response of the three-electrode system to “light” and “dark” modes that lasted 10 s each over a period of 100 s. It can be seen in Figure 4b that unusual spikes exist at the first exposure to the solar simulator and that the current density values were stabilized within 100 s. According to Chouhan et al., the spike is happening due to the diffusion of charge carriers in the first cycle. Afterwards, a diffusion layer is formed at the interface of the electrolyte and the electrode, which leads to a stable current density in the following cycles [29]. Median current density values for the bare ZnO nanoflowers, p-PCDA@ZnO, and Pt/p-PCDA@ZnO photoanodes under solar simulation are 0.084 mA/cm^2^, 0.336 mA/cm^2^, and 0.476 mA/cm^2^, respectively. Additionally, an XRD characterization of Pt/p-PCDA@ZnO before and after the PEC experiment was performed, and the results are presented in Figure S3. No significant changes were observed before and after PEC, confirming the integrity of the photoanode. Further, we compared current densities from other, similar photoanodes in Table 1. Even though the Pt/p-PCDA@ZnO has a similar current density, it has the advantage of a photosensitizing property. The absorption range of Pt/p-PCDA@ZnO is expanded to longer wavelengths of 500–650 nm, which allows it to harvest the energy from the visible range of irradiation compared to others.

**Figure 5:**
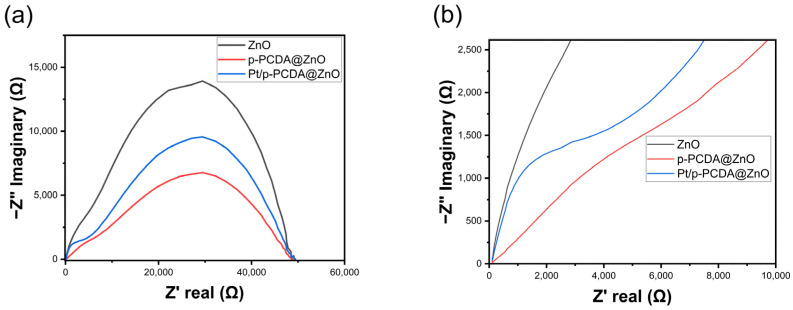
(**a**) Electrochemical impedance spectroscopy (EIS) of ZnO nanoflowers, p-PCDA@ZnO, and Pt@p-PCDA@ZnO photoanodes, and (**b**) the enlarged region (up to 10,000 Ω) where the double arcs were connected.

Even though the Pt/p-PCDA@ZnO photoanode shows outstanding PEC performance, especially for multi-interfacial electrodes, an interfacial resistance should be considered. If electrons experience high resistance at the interfaces, it may deteriorate the performance of the electrode. For a precise understanding of the interfacial charge transfer, electrochemical impedance spectroscopy (EIS) analysis was conducted. This technique can offer in-depth analysis about depletion zone thickness and charge recombination rates in the system, especially for multi-layered photoelectrodes in SWS. Multi-layered photoelectrodes that are used in PEC water-splitting are witnessed to have semicircles in EIS data [32]. Likewise, we also expected similar behavior in the Pt/p-PCDA@ZnO photoanode. Figure 5 shows the EIS data of the three photoanodes: bare ZnO nanoflowers, p- PCDA@ZnO, and Pt/p-PCDA@ZnO. As can be seen in Figure 5a, the bare ZnO nanoflower photoanode has the largest arc radius compared with those of the p-PCDA@ZnO and Pt/p-PCDA@ZnO photoanodes. The smaller the arc radius, the more efficient charge transfer; conversely, a larger arc radius means less efficient charge transfer due to a higher impedance. So, according to EIS spectra, the bare ZnO nanoflower photoanode has the poorest conductivity and the highest impedance among the three photoanodes considered in this study. The Pt/p-PCDA@ZnO photoanode shows a higher impedance compared to that of the p-PCDA@ZnO photoanode, and it can be seen from the enlarged EIS spectra in Figure 5b. In the Pt/p-PCDA@ZnO EIS data, we have a distinct arc coming from the interfacial resistance at impedances ranging from 1500 Ω to 4000 Ω. 

The PCDA@ZnO EIS data show a weak arc at impedances ranging from 1500 Ω to 7000 Ω. The arc located in a former impedance range must arise due to the Pt nanoparticles and the p-PCDA interface in the Pt/p-PCDA@ZnO photoanode. In the SWS EIS analysis of different Pt nanoparticle and ZnO nanorod interfaces in other studies [33,34], the tendency of forming arcs under 1000 Ω can be noted. Therefore, we believe that the catalytic amount of Pt nanoparticles is responsible for the arc formation in the EIS spectrum of the Pt/p-PCDA@ZnO photoanode at impedances from 500 Ω to 2500 Ω with a peak near 1000 Ω.

### 3.3. UV-Vis Measurements and Gas Evolution Tests

**Figure 6:**
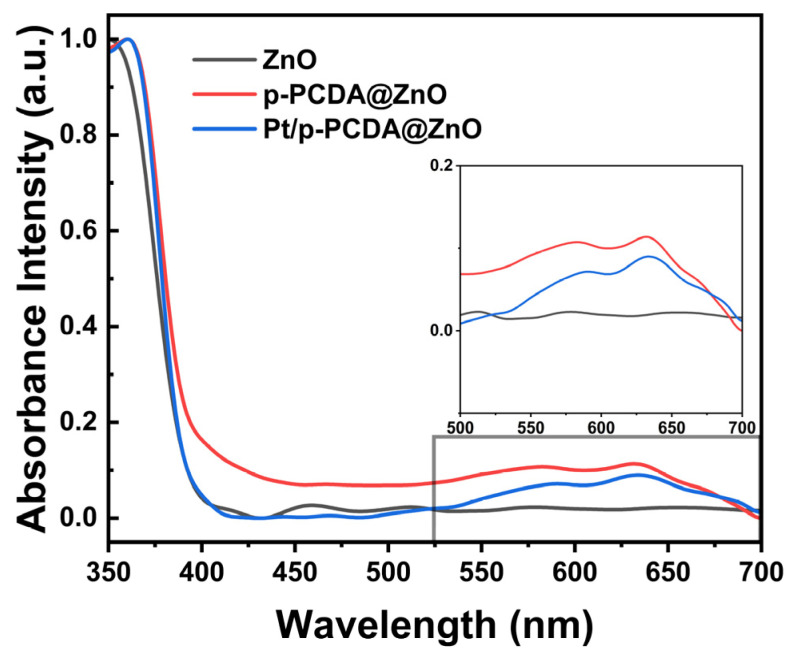
Absorbance intensity of the bare ZnO nanoflowers and p-PCDA@ZnO and Pt/p- PCDA@ZnO photoanodes at wavelengths ranging from 350 nm to 700 nm. Inset: enlarged absorbance intensities data in the visible range (wavelength range of 500~700 nm). The effect of the p-PCDA layer can be seen, with peak absorbance intensities at 589 nm and 633 nm.

The UV–visible spectroscopy measurement was performed at wavelengths from 350 nm to 700 nm to confirm the role of p-PCDA as a photosensitizer in the hybrid photoanodes. Figure 6 shows absorbance intensities of the three different photoanodes used in this study. The data show that in the wavelength region of 550~650 nm, both the p-PCDA@ZnO and Pt/p-PCDA@ZnO photoanodes have higher absorbance intensities compared to that of the bare ZnO nanoflower photoanode. The UV region of solar light contributes only ~4% in the whole solar light spectrum, and most of the solar irradiation intensity lays in the visible and infrared region. Therefore, even a small increase in the absorbance intensity of p-PCDA film in the visible range can have a significant effect on the PEC performance of photoanodes. From the UV–visible spectrum, uplifting of the p-PCDA@ZnO baseline is observed, which can originate from light scattering. Light scattering might happen due to the surface roughness or different film thicknesses. Thickness can vary since our samples are patterned and have spacing in between the ZnO nanoflowers. Moreover, for the hybrid samples, light scattering can happen because of the different refractive indices of the organic and inorganic layers [35]. Also, in the spectrum of ZnO, bands around 400–500 nm can be seen. These bands may originate from the presence of surface states. Surface states have different bandgaps compared to bulk ZnO [36]. ZnO can react with the atmosphere, resulting in varying surface states on the surface of the ZnO nanoflowers. However, after coating with p-PCDA, the surface is passivated, and hence no peak is observed around 440~500 nm. The inset box of Figure 6 shows the enlarged absorbance spectra of the three photoanodes in the visible region (the wavelength ranging from 500 nm to 700 nm). The absorbance peak of p-PCDA can be found at 589 nm and 633 nm, and a clear enhancement of the absorbance of the p-PCDA layer-coated photoanodes in the visible region can be observed. In Figure S5, UV–visible spectra of the PCDA@FTO and p-PCDA@FTO are shown. In a polymerized sample, we can see increased absorbance, with peaks near 550 nm and 650 nm. This is happening due to the formation of conjugated systems in the p-PCDA. Even though the Pt/p-PCDA@ZnO and p-PCDA@ZnO absorption peaks are slightly shifted, this still confirms that an increased output of a hybrid photoanode in PEC is due to the photosensitizing effect of p-PCDA in the visible range of radiation.

**Figure 7:**
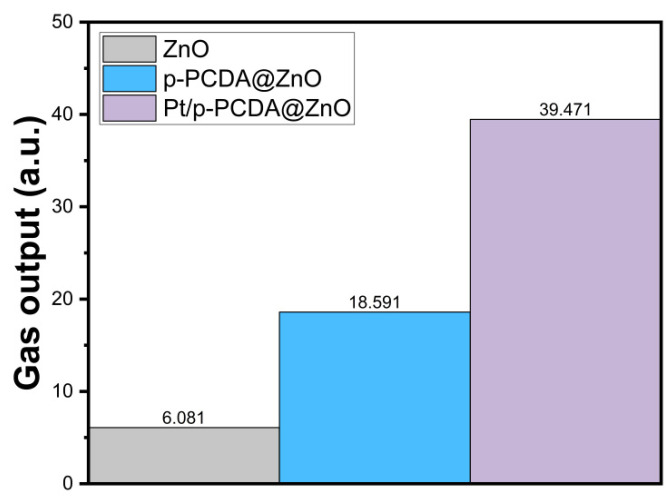
The estimated total amount of oxygen gas from the bare ZnO nanoflowers and p- PCDA@ZnO and Pt/p-PCDA@ZnO photoanodes under simulated solar irradiation of 100 mW/cm^2^ (AM 1.5) for 10 min.

We also measured how much oxygen gas can be obtained from the bare ZnO nanoflowers and p-PCDA@ZnO and Pt/p-PCDA@ZnO photoanodes under simulated solar irradiation of 100 mW/cm^2^ (AM 1.5). We measured the amount of oxygen gas, because the gases produced from the photoanode and the cathode are oxygen and hydrogen, respectively. We believe that the amount of oxygen gas released from the photoanode must be proportional to that of the hydrogen gas produced on the cathode. Therefore, it is reasonable to estimate the amount of hydrogen produced based on the amount of oxygen released. The gas evolution measurement system was the same as the PEC measurement system, which consists of three electrodes that are photoanodes, Ag/AgCl as a reference electrode, and Pt mesh as a counter electrode. The gas production performance of the three different photoanodes was recorded for 10 min, and screenshots of the end stage of the gas production are provided as Supplementary Information in Figure S6. The amount of gas produced from the photoanode was estimated by using images from the video recording. The amount of produced gas was estimated by counting the number of pixels under gas bubbles in each frame of the images. While the size of the photoanodes remains the same, the frame of the images is slightly different. Therefore, normalization was carried out for the gas output estimations. Figure 7 shows the amount of gas production (arbitrary units) within 10 min, and we can clearly see in this figure that the gas produced from Pt/p-PCDA photoanode is the highest. It is about two and six times higher than that from the p-PCDA@ZnO photoanode and the bare ZnO nanoflower photoanode, respectively. 

## 4. Conclusions

In this paper, we tried to improve the light absorption ability of the bare ZnO nanoflower-based photoanode in the PEC performance of the solar water splitting by introducing an organic monolayer of p-PCDA and a Pt nanoparticle as a co-catalyst. The comparative study of PEC performance in solar water splitting was conducted by using bare ZnO nanoflowers and p-PCDA@ZnO and Pt/p-PCDA@ZnO photoanodes. We found that the Pt/p- PCDA@ZnO photoanode showed the highest current intensity in PEC measurement and produced the largest amount of hydrogen gas due to enhanced light absorbance in the visible range from the p-PCDA layer and the Pt co-catalyst. Therefore, we can conclude that the Pt/p- PCDA@ZnO photoanode structure certainly has great potential for development in solar water splitting applications.

## Figures and Tables

**Table 1:** The list of ZnO-based hybrid photoanodes and related electrolytes, output current density, and used illumination sources in SWS.

Photoanode Type	Electrolyte	Current Density	Illumination Source	Ref.
P3HT/ZnO	0.1 M Na_2_SO_4_	0.642 mA/cm^2^ at 0.75 V	80 mW/cm^2^	[30]
TiO_2_/ZnO	0.1 M Na_2_SO_4_	1.45 mA/cm^2^ at 0.8 V	100 mW/cm^2^	[31]
BPEI@ZnO	0.5 M Na_2_SO_4_	0.25 mA/cm^2^	100 mW/cm^2^	[18]
PEDOT:PSS/CdS_s_/ZnO/WO_2_	0.2 M Na_2_S, 0.3 M Na_2_SO_4_	0.5 mA/cm^2^	100 mW/cm^2^	[19]
Pt/p-PCDA@ZnO	0.05 M Na_2_CO_3_/NaHCO_3_	0.476 mA/cm^2^ at 1.23 V	100 mW/cm^2^	This work

## Data Availability

Data can be provided by the authors upon reasonable request.

## Supplementary Materials

The following supporting information can be downloaded at: https://www.mdpi.com/article/10.3390/ma17174259/s1; Figure S1: The cross-section SEM image of p-PCDA film-coated ZnO nanorods; Figure S2: HRLV SEM images of p-PCDA@ZnO (a,b) and enlarged images of p-PCDA@ZnO (c) and Pt/p-PCDA@ZnO (d); Figure S3: XRD data of Pt/p-PCDA@ZnO before and after PEC; Figure S4: Chronoamperometry of p-PCDA@ZnO with different rounds of coatings; Figure S5: UV–visible spectra of PCDA and p-PCDA on FTO glass; Figure S6: The gas output from photoanodes.
