# Supplementary material for: PCDA/ZnO Organic–Inorganic Hybrid Photoanode for Efficient Photoelectrochemical Solar Water Splitting
Source: Materials (Basel). 2024 Aug 28;17(17):4259. doi: 10.3390/ma17174259 (PMC11396138; doi:10.3390/ma17174259)
Supplement: Supplementary file 1 [file materials-17-04259-s001.zip › materials-3159623-supplementary.pdf]

materials

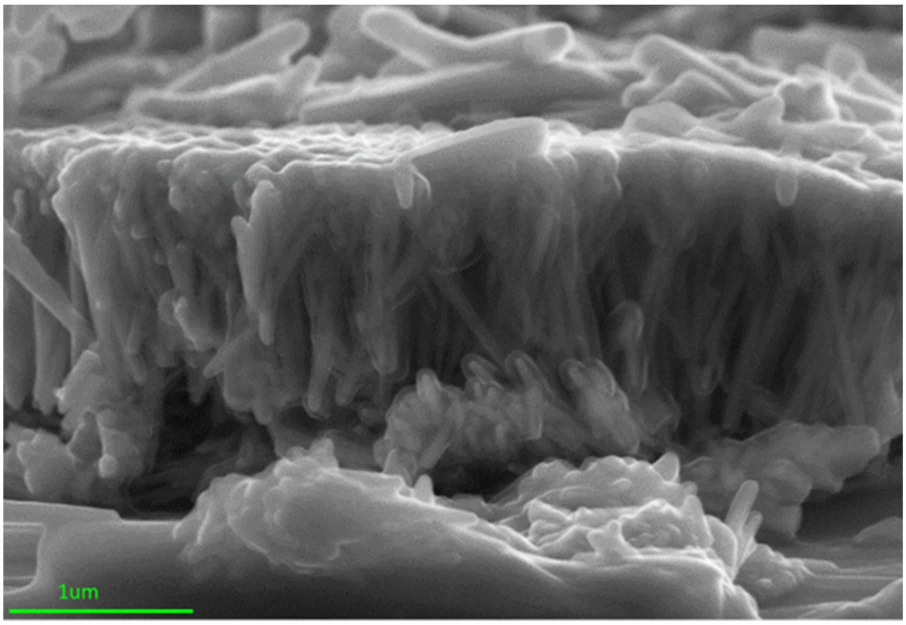

Figure S1. The cross-section SEM image of p-PCDA film coated ZnO nanorods.

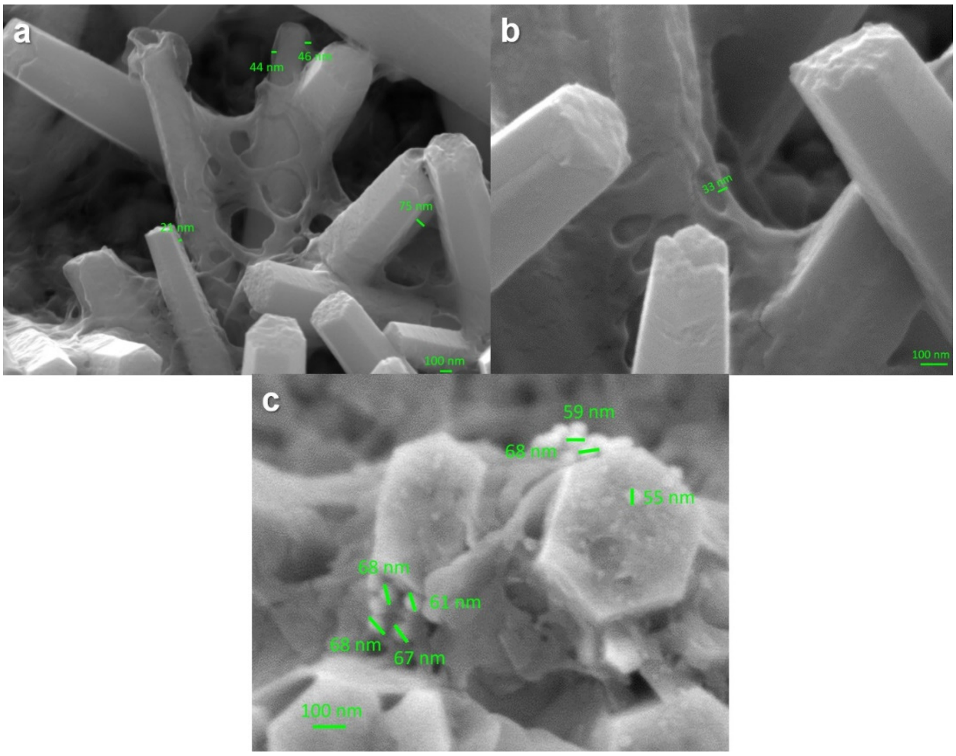

Figure S2. HRLV SEM images of p-PCDA@ZnO (a), enlarged image of p-PCDA@ZnO (b) and Pt/p-PCDA@ZnO (c) with scales.

Materials **2024**, *17*, 4259. https://doi.org/10.3390/ma17174259 https://www.mdpi.com/journal/materials

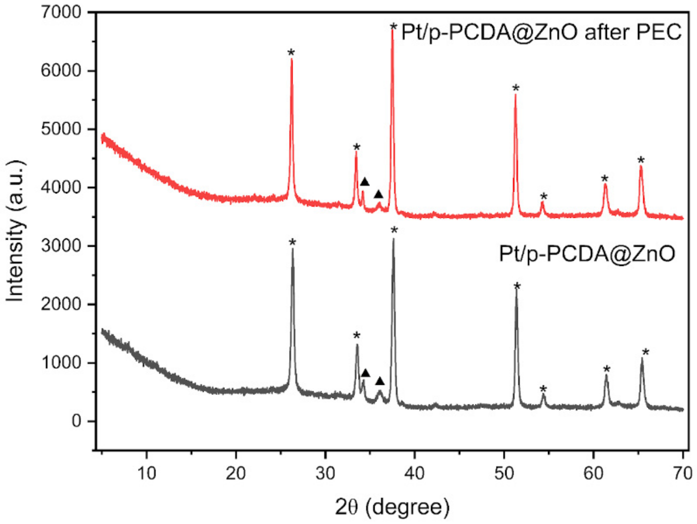

Figure S3. XRD data of Pt/p-PCDA@ZnO before and after PEC.

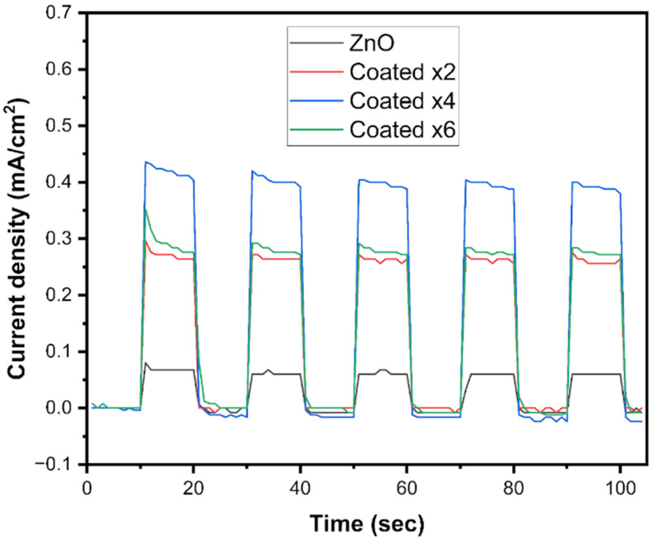

Figure S4. Chronoamperometry of p-PCDA@ZnO with different rounds of coatings.

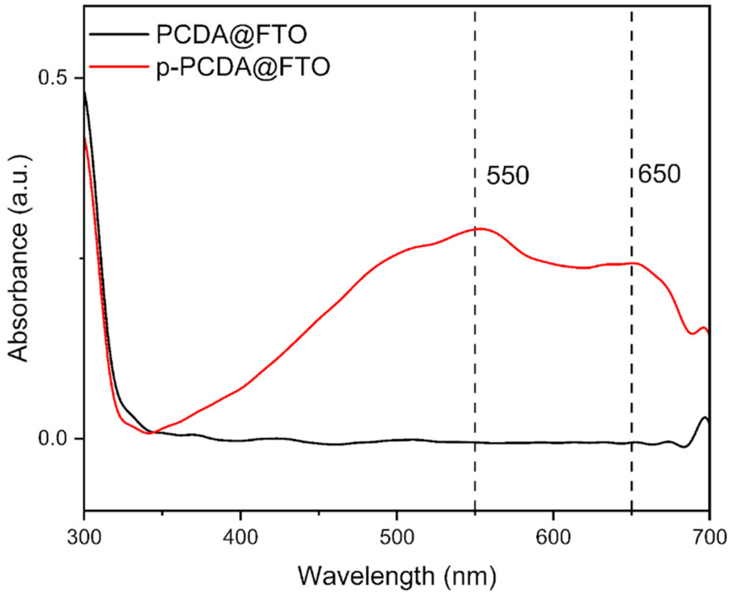

Figure S5. UV-Visible spectra of PCDA and p-PCDA on FTO glass.

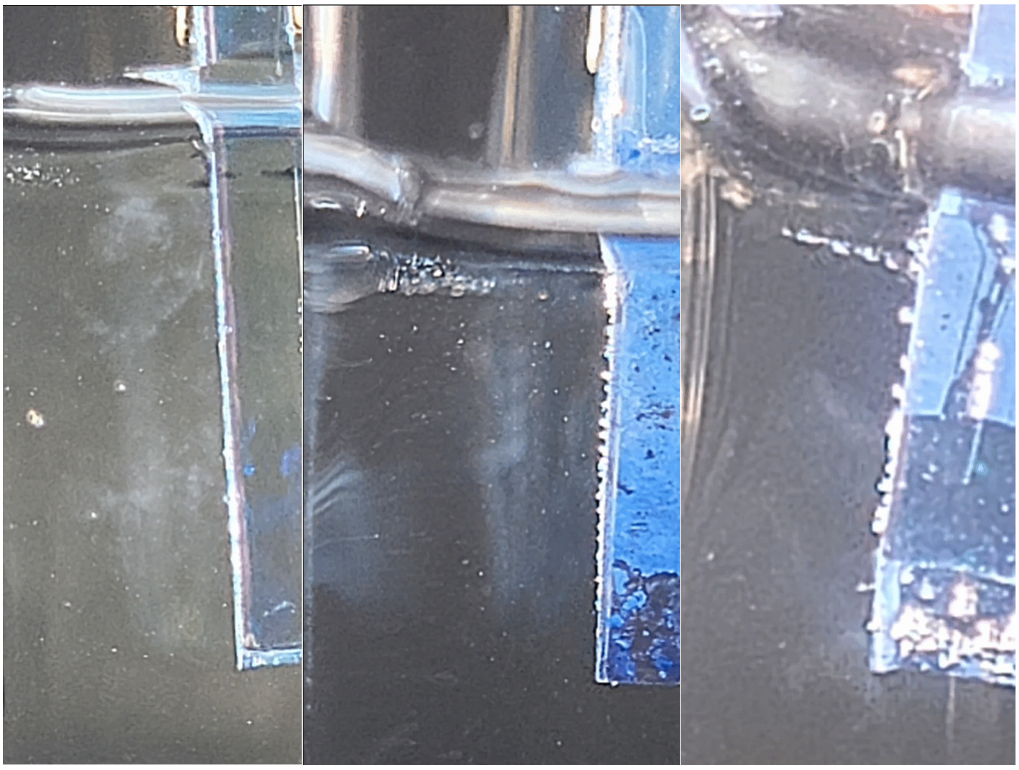

Figure S6. The gas output form photoanodes under simulated solar irradiation (AM 1.5, 100 mW/cm^2). From left to right, images of ZnO, p-PCDA@ZnO and Pt/p-PCDA@ZnO photoanodes.
